# Language, writing, and activity disorder in the autistic spectrum

**DOI:** 10.3389/fnint.2013.00042

**Published:** 2013-05-29

**Authors:** Daniel Orlievsky, Sebastián Cukier

**Affiliations:** Facultad de Psicología, Hospital Infanto Juvenil “Dra. Carolina Tobar García”, Universidad de Buenos AiresBuenos Aires, Argentina

This comment is based on our research studies related to the development of written language in persons with Pervasive Developmental Disorders (American Psychiatric Association, 1998) who lacked language, or whose language was echolalic or bizarre, limited to few words and who did not communicate by means of sign language or handwriting.

The interest of this comment is to explore the possibilities of developing communication by means of writing and to study differences between spoken and written language. In our studies we instrumented an approach focused on independent writing and we considered our conclusions only when independence was achieved. Another important point is that since it is supposed that written language is acquired after oral language it is common not to teach writing to patients with severe developmental disorders that lack language or whose language is sufficiently disturbed so as to presume lack of comprehension. However, the cases studied showed us that this strategy is possible for a series of them. It is possible to invert the order as a function of the child's capabilities and predispositions to allow for a smooth transition from written to spoken language that is tailored to the individual. Finally, we consider it interesting that some patients could develop some functional language at a much older age than previously considered possible.

To achieve the objetive we developed a “Writing Program for the Habilitation of Language.”

The approach used attempts to help the individuals to communicate by means of writing using a computer or a similar device and using, only initially, physical support (holding the hand of the subject whom we want to assist so that he can initiate the action, control impulsivity and/or perseverations due to disorders in the elaboration of complex voluntary motor actions). We start out by pointing to figures, to later move on to copying words, completing blank spaces in a sentence (predictable and unpredictable) and the highest level expected is achieving open independently written conversations.

In our approach, when physical support was necessary, we first tried to make the person write independently starting the process by copying words until they could write by themselves. Once the subjects could write independently we try to develop further language through writing, following the person's interest and trying to increase communication abilities. This is in contrast to traditional “Facilitated Communication” (FC), the technique Developed by Crossley in Australia in the 70's (Crossley, 1994), that do not necessarily promote independence in writing, which could lead to possible use of influence or induction from the facilitator (Jacobson et al., 1993).

The fundamental principles for the instrumentation of the technique are, as in FC, based on the importance that, motor disorders (apraxias or dyspraxias) may have on these types of ailments. We consider that the FC technique is efficient for certain cases and not for others. In a study criticizing classical FC, published in 1994, Carol Vazquez concludes that in the cases in which the individuals needed physical support, in general, correct responses were written only when the facilitators knew the response. However, one case described in one study (Eva) was able to respond 9 out of 10 items correctly on her own (the facilitator was unaware of the figure that Eva had been shown) (Vazquez, 1994). “While the abilities of many persons with autism are overrated, habilitation of language through writing can focus attention on those cases with speech disorders that are truly educable and can benefit from individualized educational programs.”

We agree with Vazquez in that efforts of intensive and controlled validation must be carried out in case by case studies to determine which persons would truly benefit from the technique.

Every person that entered the program was simultaneously helped by two or more researchers, with a frequency of a 30-min weekly session. During the course of the studies with more than 25 subjects between 6 and 25 years old, the process of acquirement of writing has been uneven among subjects. This enables us to consider that there may be cases in which the capacity of writing may be preexistent and may not have been identified, as well as others (not alphabetized) in which writing was constructed gradually from the strategies that were implemented. In any case, some of the children and adolescents that had no functional means of communication with others are now developing one.

In the first consistent description of “early infantile autism” published 70 years ago in “Nervous Child” journal, Kanner writes that “Eight of the eleven children acquired the ability to speak at the normal age or with some delay. Three (Richard, Herbert, Virginia) have remained ‘mute’ until today. None of the eight children who ‘speak’ have been able to use language several years to communicate meaning to others” (Kanner, 1943). In a latter study on language (Kanner and Eisenberg, 1956), of a total of 42 cases studied that were re-examined by the authors over a period of several years, 19 had not acquired language, remaining in withdrawal and showing no evolution; 23 had acquired language and among these, only 12 showed schooling capacity. “For the majority of those who achieved the development of language, there was an important difficulty to learn the correct use of pronouns and, even though they speak, none of the contents intend to have communicative value. There were verbal rituals, irrelevant expressions, repetitions, literal and inflexible use of words, questions of obsessive nature, immediate or deferred echolalia, non-initiation of conversations, as well as semantic, syntactic and pragmatic disorders, etc.”

The severity of the language disorder is the greatest difficulty for their clinical and educational progress. Some authors showed that the absence of language was the main concern expressed in neurological consultations by more than half of the parents of autistic children that are in pre-school (Tuchman et al., 1991; Soprano, 1997). While Rutter (1979) and others established that children who remain non-verbal at age 5% an unfortunate prognosis, Rappin, who coincides, refers to an exceptional case who started speaking fluently at age 10. A study of cases carried out by Rutter et al. (1967) found that 50% of individuals suffering from autism remained non-verbal at age 5 and 75% of those who spoke presented echolalia or other abnormal characteristics. In general terms it is considered that while 1 of every 5/6 individuals suffering disorders within the Autistic Spectrum never speak and remain mute all their lives others never overcome the stage of echolalia (Rappin, 1987, 1994; Cukier, 2005).

Even though language disorders within the AS and have been extensively studied by numerous authors, our research studies carried out by the “Communicational through writing habilitation Program” of the Infant Juvenile Psychiatric Hospital “Dr Carolina Tobar Garcia” and the School of Psychology of the University of Buenos Aires allows for some contributions regarding individuals that, within the autism spectrum, are among those most affected and of worse prognosis (with limited or non-functional language) (Orlievsky and Calzetta, 2004). Five subjects were able to develop written language after 14 years old, to the point of being able to hold written conversations with therapists and one of them through e-mail with relatives and started to use basic oral language too. The results of this investigation, together with the clinical description of the subjects studied can be consulted in publications of Investigation Seminars as well as in Annuals XII and XIII (Calzetta and Orlievsky, 2005; Orlievsky, 2012) and Outreach Program at the School of Psychology, University of Buenos Aires.

If we try to explain the factors that influence the acquirement of writing and the link between the development of writing with activity disorders, the contributions of Azcoaga et al. (1997) in relation to the physiopathology of language are of help. Although these author distinguishes aphasias in general from severe developmental disorders, in our opinion it is possible to explain some language disorders through aphasic mechanisms. Among these contributions we propose that abnormal forms of language inhibition might exist. The author describes the “Baillarger-Jackson phenomenon” which consists of the impossibility of a patient to pronounce a word at the moment it is requested from him, but has the ability to do so while under the effects of an emotional state. He considers the dissociation between “voluntary” and “automatic” language. Certain language functions are blocked and certain states (emotions, for example) unblock (facilitate) verbal expressions. This phenomenon, called “facilitation” is what allows some patients to produce expressions, phrases or names that cannot be emitted under the conditions of “voluntary” language (Azcoaga et al., 1997). In this same sense, the concept of facilitation enables us to explain some of the processes that we have seen in which writing, apart from emotional stimuli, has in some cases enabled the development, and the unblocking in others, firstly of written language and of oral language later on. This corroborates and corresponds to a higher psychic organization that is observed in the cases described. Cerebral cortex and other brain structures organize themselves as certain functions are performed.

Angel Rivière intends to articulate the first undifferentiated impression of lack of finality and purpose, the *absence of meaning* of the autistic conduct (Riviere, 1996). He finds an objective basis for the vague impression of “lack of meaning” provoked in us by the conduct of children with symptoms that fall within the autistic spectrum: “When those behaviors are examined objectively and rigorously encoded, we can see a lack of development of those actions that intentionally imply purpose, inherent creativity, projection towards the future, meaning in a word” (id).

What we could observe in some of our cases is that the writing modified these meaningless actions thus enabling some organization of behavior and development of language. The conducts of these patients who presented aimless wandering and racing, turning on and off of lights, hair pulling, repetition of numbers and insults, marked impulsivity, etc., were reduced after initiating the writing process thus explaining how language modulates and organizes conducts which depend on language itself. Being that these characteristics are present in the most severe cases, i.e. the ones that lack language or present severely disturbed language, it is likely that the development of language (in the referenced cases) was what allowed for regulation of behavior in semiotic terms. The point is to explain the phenomenon that we have observed by trying to understand why written language allows these processes to develop. Elizabeth Torres suggested that the machinery of muscles that we have to produce and recognize sounds may have a similar architectural foundation as that for gesturing and writing language. Thus, a proper map can be established between the two systems but it takes some time to establish that map and in this sense order matters. Normally we hear language, parse it and decode it and we talk eventually, then we write. The technique might be a way to build this map between the muscles that do the writing and the machinery to produce and interpret sounds at some stage of the learning progression of the child. It is probably different for each person so at an individual level there will be some features that you can identify yet something universal about it must exist where you achieve these across the broad spectrum and to a certain extent can lead the child to eventually speak.

Azcoaga suggests that in the aphasias the central role in the encoding/decoding of language is played by the verbal analyzer, on whose function the kinesthetic-motor verbal analyzer is dependent (Azcoaga et al., 1997). Some pathological inhibition affects the comprehension of language in variable degrees: loss of comprehension, except for some isolated words (the most consolidated ones); phrases in a context, and in the mildest degree of that inhibition, the difficulty to grasp what is most abstract and subtle in a context. These processes operate both in the child as well as in the adult. In the latter it alters the analytical-synthetic activity of the language analyzers. In the child it blocks the learning process of elocution and comprehension. Due to the auditory characteristic of oral language and to the visual characteristic of writing it is possible to presume that the auditory verbal analyzer and/or kinesthetic-motor verbal analyzer are compromised (in these cases) to a greater extent than the visual analyzer (Azcoaga et al., 1997).

We came across patients that initially could not associate the sound of the letters that were being taught but were able to incorporate them if presented in writing. Only after a certain time of learning were they able to incorporate the auditory support without need of being presented with the written letters. Just as we saw above, these cases are compatible with the idea that other brain structures organize themselves as certain functions are performed.

The approach we implemented is of low intensity, so compatible with other therapies that patients are doing, and its application is easily replicable. Although it is still to clarify the exact profile of patient that might respond to it, we think that it brings hope, particularly to older and severe patients with ASD diagnosis, to develop new communication possibilities through writing.
